# Dynamic Relationship between Sense of Agency and Post-Stroke Sensorimotor Deficits: A Longitudinal Case Study

**DOI:** 10.3390/brainsci10050294

**Published:** 2020-05-15

**Authors:** Yu Miyawaki, Takeshi Otani, Shu Morioka

**Affiliations:** 1 Graduate School of Health Science, Kio University, Kitakaturagi-gun, Nara 635-0832, Japan; 2 Research Fellow of Japan Society for the Promotion of Science, Chiyoda-ku, Tokyo 102-0083, Japan; 3 Department of Rehabilitation Medicine, Keio University School of Medicine, Shinjuku-ku, Tokyo 160-8582, Japan; 4 Department of Rehabilitation, Ishikawa Hospital, Himeji, Hyogo 671-0221, Japan; goo.goo.pt4@gmail.com; 5 Neurorehabilitation Research Center, Kio University, Kitakaturagi-gun, Nara 635-0832, Japan; s.morioka@kio.ac.jp

**Keywords:** sense of agency, post-stroke, sensorimotor deficits, misattribution, cue integration, motor control, agency judgment, self-other attribution

## Abstract

Post-stroke sensorimotor deficits impair voluntary movements. This impairment may alter a person’s sense of agency, which is the awareness of controlling one’s actions. A previous study showed that post-stroke patients incorrectly aligned themselves with others’ movements and proposed that their misattributions might be associated with their sensorimotor deficits. To investigate this hypothesis, the present study compared the agency dynamics in a post-stroke patient A (PA) with sensorimotor deficits, who rarely used her paretic upper limbs in her daily life to patient B (PB), who had a paretic upper limb with almost normal functions and activity. At the second, fourth, and eighth weeks following their strokes, PA and PB completed experiments where they performed horizontal movements while receiving visual feedback, and analyzed if the visual feedback represented their own or another’s movements. Consequently, PB made no misattributions each week; whereas, PA made incorrect self-attributions of other’s movements at the fourth week. Interestingly, this misattribution noticeably decreased at the eighth week, where PA, with an improved paretic upper limb, used her limb almost as much as before her stroke. These results suggest that the sense of agency alters according to the sensorimotor deficit severity and paretic upper limb activity.

## 1. Introduction

Stroke is one of the most common disease that causes sensorimotor deficits. Due to their sensorimotor deficits, post-stroke patients often have difficulties in controlling their intended movements or actions. This difficulty may alter the sense of agency, which is the awareness of controlling one’s actions [1,2,3]. In terms of motor control, the sense of agency is provided through a sensorimotor comparison, wherein sensory feedback is compared to its prediction based on an efference copy of the motor commands [4,5,6]. When the sensation is consistent with the internal prediction, that sensation is attributed to the self, i.e., the registration of agency [7,8]. A previous study showed the altered agency attribution in post-stroke patients with neurological dysfunctions such as apraxia [9] or anosognosia [10]; these dysfunctions are induced by damages in the parietal or premotor cortices [11,12,13] that are associated with sensorimotor comparisons [13,14]. These studies suggested that the impaired sensorimotor comparison system resulted in agency disturbances. If post-stroke patients have an impaired sensorimotor comparison system because of their sensorimotor deficits, they might make altered self-other attributions when receiving sensory feedbacks of their movements or external sensations, even if they have no neurological dysfunctions such as apraxia or anosognosia.

Our recent study [15] examined this possibility using the modified Asai’s [16] self-other attribution task where participants were required to make self-other attributions of their movements’ visual feedback. In this study [15], only patients with subcortical lesions were recruited because the lesions in the cortical regions, such as the premotor or parietal cortices, can cause neurological dysfunctions such as apraxia or anosognosia [11,12,13]. This may obscure the relationship between sensorimotor deficits and sense of agency. Participants performed horizontal tracing movements and received corresponding visual feedback via a cursor. The cursor feedback represented the participants’ actual movements or prerecorded others’ movements that were spatiotemporally unlike their actual movements. Participants were required to make self-other attributions of the cursor movement based on the spatiotemporal consistency between their actual movement and the cursor movement. The results showed that post-stroke patients with sensorimotor deficits, as compared to healthy elderly participants, significantly made more incorrect self-attributions of others’ movements. Interestingly, this misattribution was observed in the performances of both paretic and nonparalyzed upper limbs. These results led to the hypothesis that the misattributions in such patients was not due to the impaired sensorimotor comparison system, but rather due to making the self-other attribution based on a different attribution strategy than that of the healthy people.

This hypothesis was based on the cue integration theory [17,18]. According to this theory, if available sensorimotor cues, including internal prediction and sensory feedback, for the registration of agency are lesser or noisier than normal, other types of agency cues, such as thoughts [19,20,21] or beliefs [22,23], can be utilized more. Some studies on patients with schizophrenia who have impaired internal predictions [24] have supported this theory [25,26]. According to these findings, the one who has difficulties in utilizing sensorimotor cues might make altered self-other attributions due to compensatory cue processing. In terms of post-stroke sensorimotor deficits, post-stroke patients receive less or noisier sensorimotor information in their daily lives because these deficits can interfere with sensory input and reduce their paretic upper limb use [27,28]. Since they are in this satiation all the time, their attribution strategy might alter, resulting in misattributions even in the performance of their nonparalyzed upper limbs (also see the discussion in [15]). Although altered agency attribution in post-stroke patients has been examined in terms of neurological dysfunctions such as apraxia or anosognosia (e.g., [9,10,29]), few studies have examined the relationship between post-stroke sensorimotor deficits and agency disturbances in terms of the agency attribution strategy. The present study, therefore, focuses on the effects of post-stroke sensorimotor deficits on the agency attribution strategy.

If the hypothesis led by the previous study [15] is true, the self-other attribution in post-stroke patients changes according to their sensorimotor deficit severity or paretic upper limb activity. Specifically, if post-stroke patients begin to use their paretic upper limbs in their daily lives as their upper limbs recover, their misattributions might be observed less because their altered attribution strategies may be restored by receiving enough sensorimotor information. Moreover, although this hypothesis targets post-stroke patients with subcortical lesions, their misattributions may have resulted from the secondary effects of sensorimotor deficits (i.e., making sensorimotor information less or noisier) rather than the strokes themselves (i.e., subcortical lesions). If strokes can trigger misattributions, post-stroke patients should be making incorrect self-other attributions soon after their strokes. Conversely, if it were the secondary effects of sensorimotor deficits altering the attribution strategy, the misattributions would be observed after a certain period from the strokes.

To examine these possibilities, we conducted a longitudinal case study using our previous study paradigm [15] (i.e., modified Asai’s [16] self-other attribution task). The agency dynamics in two post-stroke patients were examined: Patient A (PA) and Patient B (PB); PA with sensorimotor deficits rarely used her paretic upper limb in her daily life; and PB, with very mild sensorimotor deficits, almost used his paretic upper limb normally. It was expected that PA received less or had noisier sensorimotor information than PB. We hypothesized that PA made incorrect self-other attributions after a certain period from her stroke, whereas PB did not. We also hypothesized that as PA’s paretic upper limb recovers more and as she uses her limb daily, her misattributions will no longer be observed. The present longitudinal case study examined these hypotheses.

## 2. Materials and Methods

### 2.1. Participants

**Figure 1:**
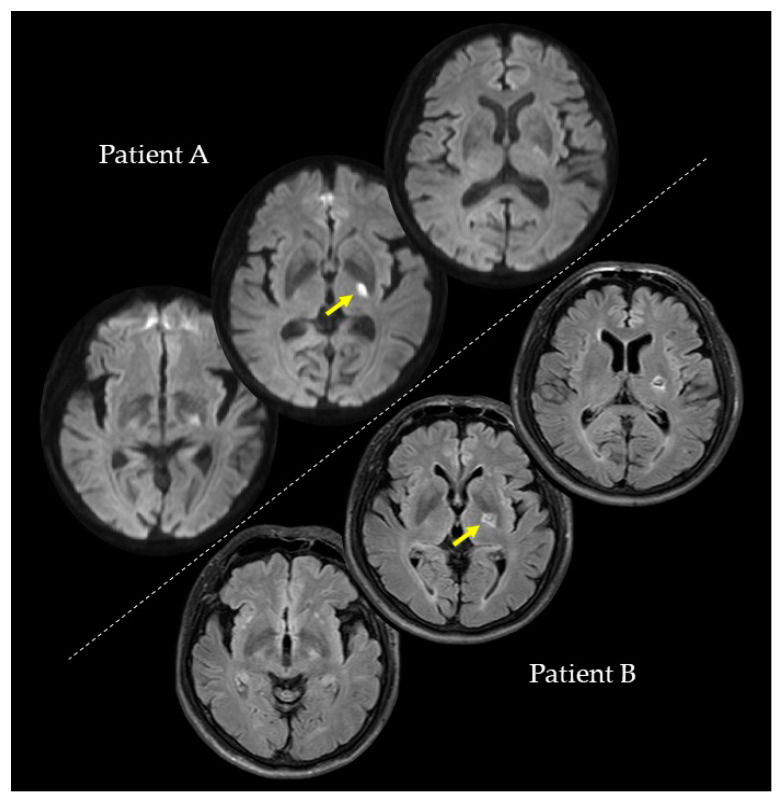
Magnetic resonance imaging scans showing the lesion sites of Patients A and B. Yellow arrows indicate the lesion sites.

We conducted the investigations of PA and PB at the second, fourth, and eighth weeks after their strokes. In the second week, PA (73 years; female), with a lesion on the left internal capsule due to an ischemic stroke (Figure 1), had a paretic right upper limb with sensorimotor deficits and rarely used her paretic upper limb in her daily life. Regarding the pre-stroke activities of PA, she did housework in general and gardening in her spare time. After the stroke, PA hoped to have improved walking functions. On the other hand, PB (59 years; male), with a lesion on the left putamen due to a hemorrhagic stroke (Figure 1), had a paretic right upper limb with very mild sensorimotor deficits and almost normally used his paretic upper limb in his daily life. Regarding the pre-stroke activities of PB, he did deskwork using a personal computer (PC). After the stroke, PB hoped to have improved hand and finger functions for PC work and writing. PA and PB were right-handed. They had no history or diagnoses of any cognitive impairments, psychiatric disorders, or neurological dysfunctions, except for their post-stroke sensorimotor deficits. PA and PB were assessed for anosognosia through a clinical interview and for neglect using the Catherine Bergego Scale. In the clinical interview for anosognosia, the clinical expert verified whether they were aware of their sensorimotor deficits and whether they could explain these deficits. A summary of the patients’ data is shown in Table 1. This study was conducted with the approval of the Ethics Committee of the Jinjukai Ishikawa Hospital (2018-1). Both participants provided written informed consent.

### 2.2. Assessments of Sensorimotor Deficits

The motor deficits of paretic upper limbs were assessed according to the Brunnstrom stage (BRS) and by using the Fugl-Meyer Assessment of upper extremity (FMA-UE) and the Simple Test for Evaluating Hand Function (STEF). Regarding the sensory deficits of paretic upper limbs, the clinical expert assessed tactile and proprioceptive sensations. In the assessment for tactile sensation, the patients were required to answer “yes” as soon as the clinical expert touched the patient’s upper limb. The number of correct answers provided from the tests was repeated five times. In the assessment for proprioceptive sensation, each of the two joints (shoulder and second metacarpophalangeal) was passively moved back and forth in a plane, and the patients reported segment orientation (i.e., up-down test). The test was repeated six times. If the patients were able to completely respond without any errors, the proprioceptive sensation was rated “intact.” If the patients were unable to respond with confidence or made one error, the proprioceptive sensation was rated as “mild.” If the patients made two or more errors, the proprioceptive sensation was rated “absent.” There were no patients whose proprioceptive sensation was rated “absent.” 

The amount and quality of paretic upper limb use in daily lives were assessed using the Motor Activity Log (MAL), which included 14 movement items, such as “brush teeth” and “pick up cup” [30,31]. MAL consisted of two subscales: Amount of use (AOU) and quality of movement (QOM). These subscales were rated on a 5-point scale (0–5) where higher scores meant better upper limb activity. In the AOU, a score of 0 indicated that the patient “did not use paretic upper limb,” and a score of 5 indicated that the patient “used paretic upper limb as often as before the stroke.” In the QOM, a score of 0 indicated that the “paretic upper limb was not used at all for that activity,” and a score of 5 indicated the patient had “the ability to use paretic upper limb for that activity was as good as before the stroke.” The average score for each subscale was calculated (Table 1). Each item data of AOU and QOM is shown in Table A1 and Table A2.

### 2.3. Apparatus

**Figure 2:**
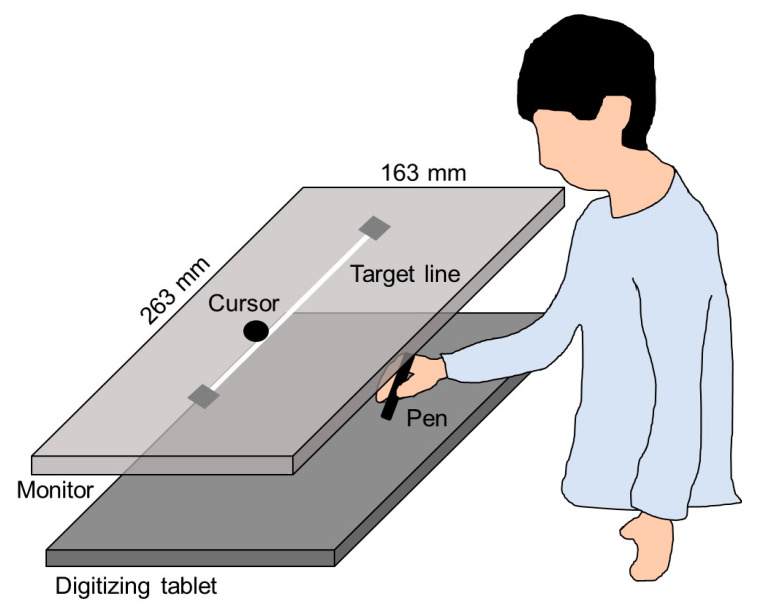
Experimental setup.

A monitor with a 60 Hz refresh rate (CF-SV7TBAQP, Panasonic) was set 20 cm vertically above a digitizing tablet (Intuos Pro Large PTH-851/K, Wacom), as shown in Figure 2. The plotting area of the monitor (263 × 163 mm) was similar to the input area of the digitizing tablet. A 230-mm straight line, which was used as the target, was horizontally displayed in the middle of the monitor. The experiment was programmed using a Hot Soup Processor 3.4 (Onion Software).

### 2.4. Procedure

The procedure performed in this study was similar to that from our previous study [15] (i.e., modified Asai’s [16] self-other attribution task) with two modifications. One of the modifications was that the experiments were conducted at the second, fourth, and eighth weeks after the stroke. Additionally, PA and PB completed the experiment using their nonparalyzed upper limb (i.e., left upper limb), contrary to the previous study where patients completed the experiment twice using their paretic and nonparalyzed upper limbs. This modification was done because we focused on the self-other attributions in a nonparalyzed upper limb use, and we needed to reduce the number of experiments to ease the burden on the patients as much as possible. The previous study confirmed that there were no significant differences in self-other judgments between the right and left upper limb uses [15]. 

First, participants placed a pen at the right side of the target line on the digitizing tablet and started moving the pen towards the left side; a computer started to count up from zero at a certain sound. After the movement was started, participants performed four cycles of horizontal movement to trace the target line such that the timing of the pen tip reaching the end of the target line (right or left side) matched an 8-second count. Movement errors, which were vertical distances between the pen position and target line, were measured as an index of motor performance. This index was not the main outcome in the present study, but it was used to confirm whether PA and PB correctly completed the movement task.

During the horizontal movements, participants received visual feedback from the cursor on the monitor (Figure 2). Regarding visual feedback, there were two conditions: SELF and OTHER. In the SELF condition, participants received the cursor feedback representing their actual pen movement. In the OTHER condition, participants received the cursor feedback representing an other’s movement that had been secretly recorded in a preliminary experiment. Participants were required to judge whether the cursor movement represented their actual or an other’s movement by referring to the online spatiotemporal consistency between their actual movement and the cursor movement. After each trial, participants verbally reported their self-other judgment on a 9-point scale ranging from 9 (completely self-movement) to 1 (completely other’s movement). Their incorrect responses meant misattributions in agency judgments.

Each time point (second, fourth, and eighth weeks) consisted of 20 trials in total, since there were 10 trials each in the SELF and OTHER conditions. Before each week’s experiment, participants were trained to be familiar with the experimental procedure through two practice sessions. In the first practice session, participants performed 20 trials with four cycles of the horizontal movement. In the second practice session, participants performed three sample trials in each of the SELF and OTHER conditions (i.e., six trials in total). After these practice sessions, the main experiment started.

## 3. Results

### 3.1. Clinical Characteristics

Table 1 shows a time course of the changes in clinical characteristics. In the second week, the BRS, FMA-UE, and STEF scores of PA were lower than those of PB. Sensory assessments showed that PA and PB had mild proprioception deficits. The MAL scores of PA were lower than those of PB, indicating that PA rarely used her paretic upper limb in almost all activities, whereas PB normally used his paretic upper limb.

In the fourth week, the FMA-UE and STEF scores were higher than those in the second week. Sensory assessments did not show any changes from those in the second week. Regarding the MAL of PA, the scores in the fourth week were higher than those in the second week, indicating that she sometimes used her paretic upper limb but performed activities with her nonparalyzed upper limb most of the time.

In the eighth week, sensory and motor assessments did not show any obvious changes, except for slight improvements of FMA-UE and STEF in PA. Regarding the MAL in PA, the scores in the eighth week were higher than those in the second week, indicating that she almost used her paretic upper limb in many activities as much as before her stroke (also see Table A1).

### 3.2. Motor Performance

**Figure 3:**
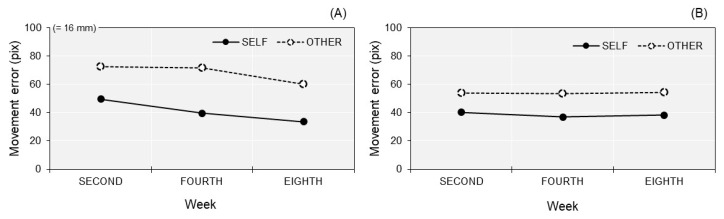
Movement errors in each experiment week (second, fourth, and eighth) for PA (**A**) and PB (**B**). For each condition (SELF and OTHER), the average movement error across cycles was calculated.

To confirm whether PA and PB completed the movement task correctly, we compared movement errors between the SELF and OTHER conditions. In this task, the movement errors in the SELF condition should be smaller than those in the OTHER condition [15,16,32]. Since differences in movement errors across cycles were not of interest, the average movement error for each condition across cycles was calculated (Figure 3). In each experiment week, the results of PA and PB showed that movement errors in the SELF condition were obviously smaller than those in the OTHER condition. Moreover, movement errors in PA were greater in the OTHER condition than that of PB.

### 3.3. Self-Other Judgment and Paretic Upper Limb Function/Activity

**Figure 4:**
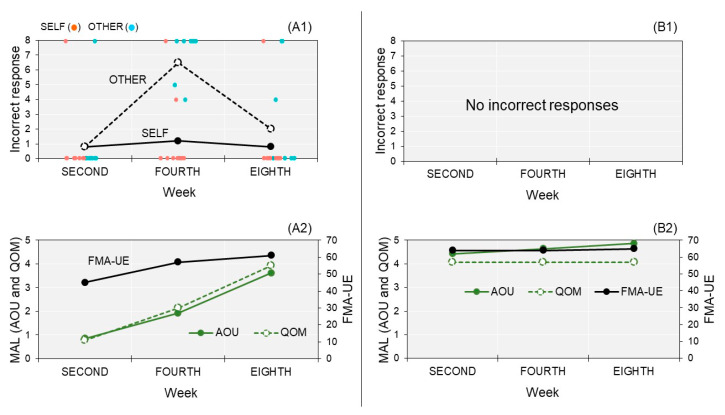
Incorrect responses (i.e., misattributions) and paretic upper limb function/activity for each experiment week (second, fourth, and eighth). **A1** and **A2** show the data of PA. **B1** and **B2** show the data of PB. In A1, the orange and blue points show the data of each trial.

To quantify misattributions for the SELF condition, the actual score of the SELF condition was subtracted from nine. To quantify misattributions for the OTHER condition, we subtracted one from the actual score of the OTHER condition. In other words, the difference between the correct and actual scores was calculated (Figure 4). The averages of their scores among trials were calculated.

In the second week, the results of self-other judgment showed that PA and PB made no obvious misattributions in the SELF and OTHER conditions. At this time, PA had more severe motor deficits than PB and rarely used her paretic upper limb in her daily life.

In the fourth week, the results showed that PA made an incorrect self-attribution of an other’s movement in the OTHER condition, whereas PB made correct self-other judgments as he did in the second week. For the clinical characteristics of PA, her motor deficits and paretic upper limb activity in the fourth week improved more than those in the second week; however, she still did not use her paretic upper limb enough in her daily life.

In the eighth week, the results showed that the incorrect self-attribution in PA obviously decreased from that in the fourth week. PB made correct self-other judgments, as he did in the second and fourth weeks. For the clinical characteristics of PA, her motor deficits in the eighth week slightly improved more than those in the fourth week, and she used her paretic upper limb in many activities almost as much as before her stroke (also see Table A1).

## 4. Discussion

This longitudinal case study examined the agency dynamics of PA, who had sensorimotor deficits and rarely used her paretic upper limb in her daily life, as compared to PB, who had very mild sensorimotor deficits and almost normally used his paretic upper limb. Consequently, PA and PB made no misattributions in a self-other attribution task at the second week; however, PA made an incorrect self-attribution of an other’s movement at the fourth week, whereas PB did not. Interestingly, this misattribution of PA noticeably decreased at the eighth week where she used her paretic upper limb in many actions almost as much as before her stroke. Regarding the results of motor performance, the difference in movement errors between the SELF and OTHER conditions was observed in each experiment week. This result replicates that of the previous studies [15,16,32], suggesting that PA and PB correctly completed the movement tasks, just like the participants in their previous studies did. Although movement errors of PA were greater in the OTHER condition than those of PB, there were no obvious differences in movement errors across the second, fourth, and eighth weeks in the performances of PA and PB. Therefore, the results of motor performance were unlikely to explain the misattribution changes of PA. The agency dynamics observed in the present study should be discussed in terms of sensorimotor deficits and its secondary effects.

The misattribution of PA was observed only in the OTHER condition. This result is consistent with the finding of the previous study that suggested that the misattribution in post-stroke patients with subcortical strokes is associated with their sensorimotor deficits [15]. The spatiotemporal motor properties of others’ movements are unlike those of one’s actual movement [16]. Therefore, if one made a self-other attribution based on spatiotemporal consistency (i.e., sensorimotor cues) between their actual and others’ movements, one would not attribute others’ movements to oneself (i.e., no misattributions). This is a normal attribution strategy because many studies have shown that the registration of agency is based on sensorimotor cues [16,33,34,35]. Conversely, post-stroke patients with sensorimotor deficits might have an altered attribution strategy. The previous study proposed the hypothesis that post-stroke patients with sensorimotor deficits can make a self-other attribution based on the consistency between their thoughts (or intentions) and sensory feedback rather than sensorimotor cues [15]. In the present study paradigm, they received the horizontal cursor movement in the same manner as the participants’ intended movement when the participants intended and performed the horizontal movement. Since the participants’ thoughts or intentions can match the cursor movements in this task, the attribution strategy based on their cognitive cues may produce self-attributions of the cursor feedback, even though its feedback represented an other’s movement [15]. Importantly, such altered attribution strategy might be associated with sensorimotor deficits because PB, with very mild sensorimotor deficits, made no misattributions in each experiment week.

PA made an incorrect self-attribution of an other’s movement in the fourth week, but not in the second week. This result supports the possibility that the altered self-other attribution is due to the secondary effects of sensorimotor deficits rather than the subcortical lesions. In the fourth week, the motor deficits of PA improved more than in the second week; however, she made a misattribution. Therefore, the motor deficits themselves might not have directly produced an incorrect self-attribution. Even if sensorimotor deficits of individuals’ upper limb resolve, they would not receive enough sensorimotor information if they do not move their upper limb and there is less motor output in their daily life. In the fourth week, PA only used her paretic upper limb sometimes. Under such conditions, PA may have gradually begun to depend on cognitive agency cues, such as thought or intention, for the registration of agency to compensate for the lesser sensorimotor information. Consequently, PA may have made an incorrect self-attribution of another’s movement in the fourth week because of the altered attribution strategy. This explanation is one of many possibilities. A further study is needed to investigate why PA made no misattributions in the second week, despite having moderate sensorimotor deficits and rarely using her paretic upper limb in many activities. Importantly, the incorrect self-attribution in PA decreased in the eighth week. In this time, PA used her paretic upper limb in many actions almost as much as before her stroke. These results suggest that a sense of agency may alter according to the sensorimotor deficit severity and paretic upper limb activity. Therefore, the present study promotes the need to further investigate the hypothesis that the misattributions in post-stroke patients with subcortical strokes resulted from the altered attribution strategy [15].

The present study showed the agency dynamics in PA and PB by investigating their sensorimotor deficits and paretic upper limb activity. For a future longitudinal study with a large sample size where implementation is extremely costly and consumes time, this finding may aid in determining the experimental procedures that can be used, such as which points in time to conduct the experiments and what clinical assessments to measure. However, several limitations of this study should be noted. First, PA and PB experienced strokes of different pathophysiological types; PA had an ischemic stroke, whereas PB had a hemorrhagic stroke. Considering that PA was 14 years older than PB, the clinical status of PA might have been potentially different from that of PB. A study with a large sample size might address these confounding effects. Although this study recruited patients based on lesion locations (i.e., PA and PB had subcortical lesions that were in similar locations), further studies are needed to investigate the impact of stroke pathophysiological types and age differences on the agency dynamics. Second, the experiments were conducted at only three time points. Therefore, a detailed time course in agency dynamics according to sensorimotor deficits and paretic upper limb activity remains unclear. In addition, the present study did not examine agency dynamics until the second week after the stroke. To further investigate the impact of strokes themselves, the study comparing a sense of agency soon after the stroke to that of pre-stroke is needed. Although the present study supports the hypothesis of the previous study [15], our hypothesis needs further investigations. The relationship between self-other attribution in post-stroke patients and cognitive agency cues should be investigated. Through these investigations, future research is expected to elucidate the causes of misattributions in post-stroke patients and its effects on their motor control, cognitive processing, or daily life actions.

## 5. Conclusions

The present longitudinal case study suggests that the sense of agency alters according to the sensorimotor deficit severity and paretic upper limb activity. For the registration of agency, the cue integration theory predicts that various self-other attribution strategies are selected according to a given situation or context [17,18,36,37]. Based on this theory, the previous study proposed that the misattributions in post-stroke patients with subcortical strokes resulted from the altered attribution strategy because of less available sensorimotor information for the registration of agency [15]. The present study produces the necessity to further investigate this hypothesis.

## Figures and Tables

**Table 1:** Patients’ clinical characteristics.

		Patient A			Patient B		
		SECOND	FOURTH	EIGHTH	SECOND	FOURTH	EIGHTH
MAL	AOU	0.85	1.92	3.62	4.43	4.64	4.86
	QOM	0.77	2.15	3.92	4.07	4.07	4.07
BRS (upper limb)		5	5	5	6	6	6
FMA (upper extremity)	Total score	45	57	61	64	64	65
	Shoulder Elbow Forearm	30	33	36	35	35	35
	Wrist	5	8	8	10	10	10
	Hand	8	14	14	14	14	14
	Coordination Speed	2	2	3	5	5	6
STEF		30	72	74	96	96	96
Tactile sensation (upper limb)		5/5	5/5	5/5	4/5	4/5	4/5
Proprioceptive sensation (upper limb)	Shoulder	Intact	Intact	Intact	Intact	Intact	Intact
	MCP	Mild	Mild	Mild	Mild	Mild	Mild
CBS (objective)		0	0	0	0	0	0
MMSE		30	30	30	30	30	30

MAL: Motor Activity Log; AOU: Amount of use; QOM: Quality of movement; BRS: Brunnstrom stage; FMA: Fugl-Meyer assessment; STEF: Simple Test for Evaluating Hand Function; MCP: Second Metacarpophalangeal joint; CBS: Catherine Bergego scale; MMSE: Mini-Mental State Examination.

## Appendix A

**Table A1:** Each item of amount of use (AOU) and quality of movement (QOM) for Patient A.

	Amount of Use			Quality of Movement		
	SECOND	FOURTH	EIGHTH	SECOND	FOURTH	EIGHTH
Hold book	1	2	4	1	3	4
Use towel	2	2	4	1	3	5
Pick up glass	1	2	4	1	2	4
Brush teeth	0	2	3	0	3	3
Shave/Make-up	n/a	n/a	n/a	n/a	n/a	n/a
Open door with key	0	1	3	0	1	3
Write/Type	0	2	3	0	1	3
Steady self	1	3	4	1	3	4
Put arm through clothing	3	3	4	3	4	5
Carry object	0	1	4	0	2	5
Grasp fork/spoon	2	2	4	2	2	5
Comb hair	0	2	3	0	1	3
Pick up cup	1	2	4	1	2	4
Button clothes	0	1	3	0	1	3

Patient A had no opportunity for the activity, “Shave/Make-up”.

**Table A2:** Each item of AOU and QOM for Patient B.

	Amount of Use			Quality of Movement		
	SECOND	FOURTH	EIGHTH	SECOND	FOURTH	EIGHTH
Hold book	5	5	5	4	4	4
Use towel	5	5	5	4	4	4
Pick up glass	5	5	5	5	5	5
Brush teeth	5	5	5	4	4	4
Shave/Make-up	4	4	5	3	3	3
Open door with key	3	4	5	3	3	3
Write/Type	3	4	4	3	3	3
Steady self	5	5	5	5	5	5
Put arm through clothing	5	5	5	5	5	5
Carry object	5	5	5	4	4	4
Grasp fork/spoon	4	5	5	4	4	4
Comb hair	4	4	5	4	4	4
Pick up cup	5	5	5	5	5	5
Button clothes	4	4	4	4	4	4
